# Clinical significance of YAP and androgen receptor expression in osteosarcoma: Evidence from a retrospective cohort study

**DOI:** 10.1097/MD.0000000000045910

**Published:** 2026-01-09

**Authors:** Juan Wang, Ping Xiang, Chuanqi Zou, Jun Wang

**Affiliations:** a Chongqing Preschool Education College, Chongqing, China; b Department of Orthopaedics, Banan Hospital of Chongqing Medical University, Chongqing, China; c Department of Joint Surgery, People’s Hospital of Chongqing Liang Jiang New Area, Chongqing, China.

**Keywords:** androgen receptor, immunohistochemistry, osteosarcoma, prognostic biomarker, progression-free survival, YAP protein

## Abstract

This study investigates the expression of Yes-associated protein (YAP) and androgen receptor (AR) in osteosarcoma and assess their prognostic significance. A retrospective cohort of 100 osteosarcoma patients and 30 adjacent normal tissues was analyzed by immunohistochemistry. Expression levels were correlated with clinicopathological features and progression-free survival. High expression of YAP (65%) and AR (60%) was significantly more frequent in osteosarcoma than in normal tissues. Both markers were associated with advanced stage and distant metastasis, and their co-overexpression predicted the shortest median progression-free survival (9 months). Interaction analysis confirmed a synergistic effect of YAP and AR on poor prognosis. YAP and AR are frequently co-overexpressed in osteosarcoma and jointly contribute to tumor aggressiveness and unfavorable outcomes. Their combined evaluation may serve as a novel prognostic indicator and potential therapeutic target.

## 1. Introduction

Osteosarcoma is a malignant tumor that primarily arises in bone and is one of the most common malignancies among adolescents and young adults.^[1]^ Despite advances in treatment modalities such as surgery and chemotherapy, the prognosis for patients with advanced or metastatic osteosarcoma remains poor, with high recurrence rates and a low 5-year survival rate. This stark reality underscores the urgent need for novel prognostic biomarkers and therapeutic targets to improve patient survival.^[2,3]^ In recent years, although numerous molecular mechanisms have been implicated in the development and progression of osteosarcoma, its exact pathogenesis remains incompletely understood.

Yes-associated protein (YAP), a key effector of the Hippo signaling pathway, has garnered increasing attention for its role in cancer biology.^[4]^ By regulating gene expression, YAP influences cellular proliferation, survival, and migration, and has been shown to promote tumor progression in various malignancies.^[5]^ Specifically in osteosarcoma, YAP overexpression has been associated with malignant features, invasiveness, and metastasis; however, its role in clinical prognosis has not been thoroughly investigated.^[6]^

Alongside YAP, androgen receptor (AR) is also gaining recognition for its role in cancer. Traditionally known for its involvement in hormone-dependent tumors such as prostate cancer, recent studies suggest that AR can promote the invasiveness of osteosarcoma cells through non-classical signaling pathways such as PI3K/AKT and WNT/β-catenin, indicating its potential oncogenic role may be independent of the hormonal microenvironment.^[7,8]^ AR activation is believed to contribute to tumor growth and metastasis, yet its precise mechanism of action in osteosarcoma remains unclear.^[9]^ Notably, YAP and AR have demonstrated synergistic oncogenic effects in other malignancies such as liver and breast cancer, but their interaction and clinical significance in osteosarcoma remain unexplored.^[10,11]^

Given the current lack of research on the combined role of YAP and AR in osteosarcoma, this study aims to fill that gap. By analyzing the expression patterns of YAP and AR in osteosarcoma tissues and exploring their associations with clinicopathological features, this study seeks to determine whether a synergistic interaction exists between the two, jointly promoting tumor progression and metastasis. We hypothesize that high expression of YAP and AR may be associated with advanced tumor stage, metastasis, and poorer progression-free survival (PFS), making them valuable prognostic indicators and potential therapeutic targets in osteosarcoma. This study aims to provide new insights into the molecular mechanisms of osteosarcoma and a theoretical basis for developing new treatment strategies.

## 2. Materials and methods

### 2.1. Data information

This study was approved by the Ethics Committee of Banan Hospital of Chongqing Medical University. This study is a retrospective analysis, involving 100 patients with osteosarcoma treated at our hospital between January 2018 and February 2020. It includes pathological slides of 100 osteosarcoma cases along with corresponding patient information, and 30 pathological slides of adjacent normal bone tissues.

Inclusion criteria:

(1) Case group: Age between ≥ 10 and ≤ 70 years; Pathological slides from initial diagnosis without any prior antitumor treatments (e.g., surgery, chemotherapy, radiotherapy); complete clinicopathological data available (tumor stage, metastatic status, pathological subtype) and follow-up data (PFS); formalin-fixed, paraffin-embedded tumor tissue samples of sufficient quality for immunohistochemical staining.
(2) Control group: Normal bone tissues located > 5 cm from the tumor margin, obtained from osteosarcoma patients.

Exclusion criteria:

(1) Case group: Presence of other malignancies or systemic immune diseases; Patients who received neoadjuvant chemotherapy or radiotherapy prior to surgery (as this may affect YAP/AR expression); poor-quality tissue samples (e.g., necrosis rate > 50% or improper fixation); lost to follow-up or follow-up duration < 12 months (which may affect the reliability of survival analysis).
(2) Control group: Presence of bone metabolic diseases (e.g., osteoporosis, bone infection).

### 2.2. Grouping criteria and immunohistochemistry methods

Samples in this study were grouped based on the protein expression levels of YAP and AR, following the procedures outlined below:

#### 2.2.1. Immunohistochemical (IHC) staining

All osteosarcoma and normal control tissue samples were formalin-fixed and paraffin-embedded sections (4 μm thick). After deparaffinization with xylene and rehydration through graded ethanol, antigen retrieval was performed using citrate buffer (pH 6.0) under high pressure at 121°C for 2 minutes. Endogenous peroxidase activity was blocked with 3% hydrogen peroxide (H₂O₂) for 10 minutes, and nonspecific binding sites were blocked with 5% bovine serum albumin for 30 minutes. Primary antibody incubation was conducted overnight at 4°C in a humidified chamber using the following conditions: YAP (CST #14074, 1:200) and AR (Abcam ab108341, 1:150). Secondary antibody incubation employed HRP-conjugated goat anti-rabbit immunoglobulin G (ZSGB-BIO, PV-6001). After 3,3′-diaminobenzidine chromogenic development, hematoxylin was used for counterstaining, and the slides were mounted using neutral resin.

#### 2.2.2. Double-blind scoring and H-score calculation

Staining results were independently evaluated by 2 experienced pathologists in a double-blind manner. The H-score was calculated by combining staining intensity (scored as 0–3 for none/weak/moderate/strong) with the percentage of positive cells (0–100%). The formula used for H-score calculation is:


$$\begin{array}{l} H\text{-}score=(1\times {\;\%\;}_{weak})+(2\times {\%}_{moderate})+(3\times {\%}_{strong}) \end{array}$$

Inter-observer consistency was validated using the intraclass correlation coefficient = 0.85, *P* < .001.

#### 2.2.3. Expression grouping definitions

YAP high expression group: H-score ≥ 150 (65 cases, 65.0%); low expression group: H-score < 150 (35 cases, 35.0%).

AR high expression group: Same threshold applied (H-score ≥ 150, 60 cases, 60.0%); low expression group: H-score < 150 (40 cases, 40.0%).

Combined grouping: Based on dual marker expression status, patients were divided into 4 groups: YAP⁺/AR⁺ (45 cases), YAP⁺/AR⁻ (20 cases), YAP⁻/AR⁺ (15 cases), YAP⁻/AR⁻ (20 cases). Threshold selection was based on preliminary validation (patients with H-score ≥ 150 had a fourfold increased risk of metastasis compared to those with < 150).

#### 2.2.4. Quality control

Each batch of experiments included positive controls (prostate cancer tissue known for high YAP/AR expression) and negative controls (phosphate-buffered saline instead of primary antibody). Samples with inconsistent staining intensity (approximately 5%) were reevaluated and consensus was reached.

### 2.3. Data collection

All patient clinical data, including age, sex, tumor stage, metastasis status, and pathological type, were obtained from the electronic medical record system and verified by designated personnel.

#### 2.3.1. Demographic information

Age and sex data were collected from the electronic medical record system and verified by assigned personnel.

#### 2.3.2. Tumor characteristics

#### Staging

Tumor staging was determined based on the American Joint Committee on Cancer 8th edition criteria, obtained from pathology reports and imaging examinations, and categorized as Stage I–II (n = 40) and Stage III–IV (n = 60).

#### 2.3.3. Metastatic status

Metastasis was confirmed through clinical follow-up records and imaging data. Based on the presence of distant metastasis at initial diagnosis, patients were divided into 2 groups: no distant metastasis (n = 70) and distant metastasis (n = 30).

#### 2.3.4. Pathological subtype

The osteosarcoma subtype was determined from pathological slides and classified into osteoblastic (n = 60) and non-osteoblastic (n = 40) groups.

#### 2.3.5. Treatment information

#### Surgical method

All patients underwent surgical treatment, including limb-salvage surgery and amputation, with the specific procedure determined based on pathological and imaging evaluations.

#### 2.3.6. Chemotherapy regimen

For patients receiving chemotherapy, the regimen was recorded. Common regimens included Cisplatin/Adriamycin combination therapy. If a patient did not receive chemotherapy, it was also documented accordingly.

#### 2.3.7. Follow-up data

The overall follow-up period for the included patients ranged from 12 to 50 months, with a median follow-up time of 24 months. Patients with follow-up <12 months were excluded to ensure reliability of survival analysis.

PFS: PFS was defined as the time from diagnosis to radiographic progression (according to Response Evaluation Criteria in Solid Tumors 1.1 criteria) or death.

#### 2.3.8. Loss to follow-up exclusion

Patients with a follow-up time less than 12 months or missing key outcome events (e.g., death, radiographic progression) were excluded from the analysis.

### 2.4. Statistical analysis

Statistical analysis was conducted using Statistical Package for the Social Sciences 26.0 and R 4.3.1, with a 2-sided *P*-value < .05 considered statistically significant. Categorical variables were expressed as frequency (percentage), and intergroup comparisons were performed using the Chi-square test or Fisher’s exact test (when expected frequencies were < 5). Continuous variables (e.g., H-score) were assessed for normality; normally distributed data were presented as mean ± standard deviation (independent-sample *t*-test), while non-normally distributed data were presented as median (interquartile range) and analyzed using the Mann–Whitney *U* test. The correlation between YAP and AR expression was assessed using Spearman rank correlation coefficient. Subgroup analyses were stratified by sex, metastasis status, and other factors. Survival analysis was performed using the Kaplan–Meier method (Log-rank test). For prognostic evaluation, the Cox proportional hazards model was applied, adjusting for age, sex, tumor stage, metastasis status, pathological subtype, as well as clinical treatment factors including surgical method and chemotherapy regimen, with hazard ratios (HR) and 95% confidence intervals (CI) reported. The combined expression effect was validated through grouped survival analysis and interaction term testing (YAP × AR).

## 3. Results

### 3.1. YAP and AR are significantly upregulated in osteosarcoma tissues

The expression levels of YAP and AR were significantly higher in osteosarcoma tissues compared to normal bone tissues. Immunohistochemical analysis showed that the high expression rate of YAP in osteosarcoma tissues was 65.0% (65/100), significantly higher than that in normal tissues (20.0%, 6/30, *P* = .001). The mean H-score (180.5 ± 45.2 vs 90.3 ± 25.1, *P* < .001) further confirmed the aberrant activation of YAP in tumor tissues. Similarly, AR exhibited a high expression rate of 60.0% (60/100) in osteosarcoma, markedly higher than that in normal tissues (10.0%, 3/30, *P* < .001), and its mean H-score (170.8 ± 40.8 vs 80.6 ± 20.5, *P* < .001) also showed a significant difference. These findings indicate a coordinated overexpression of YAP and AR in osteosarcoma, suggesting that both may jointly contribute to tumor development and progression (Table 1).

**Table 1:** Expression levels of YAP and AR in osteosarcoma tissue.

Indicator	Osteosarcoma tissue (n = 100)	Normal bone tissue (n = 30)	*P*-value
YAP expression			
High expression	65 (65.0%)	6 (20.0%)	.001
Low expression	35 (35.0%)	24 (80.0%)	
Average H-score	180.5 ± 45.2	90.3 ± 25.1	<.001
AR expression			
High expression	60 (60.0%)	3 (10.0%)	<.001
Low expression	40 (40.0%)	27 (90.0%)	
Average H-score	170.8 ± 40.8	80.6 ± 20.5	<.001

AR = androgen receptor, YAP = Yes-associated protein.

### 3.2. High expression of YAP/AR is significantly associated with advanced and metastatic osteosarcoma

High expression of YAP and AR was significantly associated with aggressive clinical features of osteosarcoma (Table 2). Among patients with advanced-stage disease (Stage III–IV), the proportion of YAP high expression was markedly higher compared to early-stage (Stage I–II) patients (78.3% vs 45.0%, *P* = .003). Similarly, AR high expression was more prevalent in advanced-stage patients (66.7% vs 50.0%, *P* = .02).

**Table 2:** Correlation analysis of YAP/AR expression with clinical and pathological characteristics.

Clinical feature	Group	YAP high expression (n = 65)	YAP low expression (n = 35)	*P*-value	AR high expression (n = 60)	AR low expression (n = 40)	*P*-value
Age	≤20 yr (n = 55)	38 (69.1%)	17 (30.9%)	.32	32 (58.2%)	23 (41.8%)	.42
	>20 yr (n = 45)	27 (60.0%)	18 (40.0%)		28 (62.2%)	17 (37.8%)	
Gender	Male (n = 60)	42 (70.0%)	18 (30.0%)	.15	40 (66.7%)	20 (33.3%)	.08
	Female (n = 40)	23 (57.5%)	17 (42.5%)		20 (50.0%)	20 (50.0%)	
Tumor stage	Stage I-II (n = 40)	18 (45.0%)	22 (55.0%)	.003	20 (50.0%)	20 (50.0%)	.02
	Stage III-IV (n = 60)	47 (78.3%)	13 (21.7%)		40 (66.7%)	20 (33.3%)	
Metastasis status	No metastasis (n = 70)	38 (54.3%)	32 (45.7%)	<.001	35 (50.0%)	35 (50.0%)	.01
	Metastasis (n = 30)	27 (90.0%)	3 (10.0%)		25 (83.3%)	5 (16.7%)	
Pathological type	Osteoblastic (n = 60)	45 (75.0%)	15 (25.0%)	.02	40 (66.7%)	20 (33.3%)	.1
	Other types (n = 40)	20 (50.0%)	20 (50.0%)		20 (50.0%)	20 (50.0%)	

AR = androgen receptor, YAP = Yes-associated protein.

In metastatic patients, the rates of high YAP and AR expression were 90.0% and 83.3%, respectively, both significantly higher than in the non-metastatic group (54.3%, *P* < .001 for YAP; 50.0%, *P* = .01 for AR). Additionally, the high expression rate of YAP was significantly greater in osteoblastic osteosarcoma compared to other subtypes (75.0% vs 50.0%, *P* = .02), whereas AR expression showed no significant difference across pathological subtypes (*P* = .1).

There was no significant association between YAP/AR expression and age (YAP: *P* = .32; AR: *P* = .42) or sex (YAP: *P* = .15; AR: *P* = .08). These results suggest that the high expression of YAP and AR may primarily drive tumor progression and metastasis, rather than being dependent on age or sex.

### 3.3. Synergistic interaction between YAP and AR is significantly enhanced in metastatic and advanced osteosarcoma

YAP and AR expression showed a significant positive correlation in osteosarcoma, with the strength of this association varying by clinical characteristics (Table 3). In the overall cohort, YAP and AR expression were moderately positively correlated (Spearman *R* = 0.52, *P* < .001). This correlation was markedly stronger among metastatic patients (*R* = 0.75, *P* < .001), suggesting a potential synergistic role in promoting tumor metastasis. Subgroup analysis revealed that the strong positive correlation between YAP and AR was especially prominent in male patients (*R* = 0.60, *P* < .001) and those with advanced-stage disease (Stage III–IV, *R* = 0.65, *P* < .001). In contrast, the association was weaker in female patients (*R* = 0.35, *P* = .028) and non-metastatic patients (*R* = 0.30, *P* = .012).

**Table 3:** Correlation analysis between YAP/AR expression and clinical features.

Group	Sample size (n)	Correlation coefficient (*r*)	*P*-value	Direction and strength of correlation
Overall cohort	100	0.52	<.001	Moderate positive correlation
Gender				
Male	60	0.6	<.001	Strong positive correlation
Female	40	0.35	.028	Weak positive correlation
Metastasis status				
Metastatic patients	30	0.75	<.001	Strong positive correlation
Non-metastatic patients	70	0.3	.012	Weak positive correlation
Age				
≤20 yr	55	0.5	<.001	Moderate positive correlation
>20 yr	45	0.55	<.001	Moderate positive correlation
Tumor stage				
Stage I–II	40	0.25	.12	No significant correlation
Stage III–IV	60	0.65	<.001	Strong positive correlation

AR = androgen receptor, YAP = Yes-associated protein.

Although both age subgroups (≤20 years and > 20 years) showed moderate positive correlations (*R* = 0.50 and 0.55, both *P* < .001), no significant correlation was observed in early-stage patients (Stage I–II, *R* = 0.25, *P* = .12). These findings suggest that the synergistic interaction between YAP and AR may play a dominant role during the later stages of tumor progression and in the metastatic process.

### 3.4. High expression of YAP and AR predicts significantly shortened progression-free survival

The median follow-up duration of the study cohort was 24 months (range: 12–50 months), which provided sufficient observation time to evaluate PFS.

**Figure 1.**
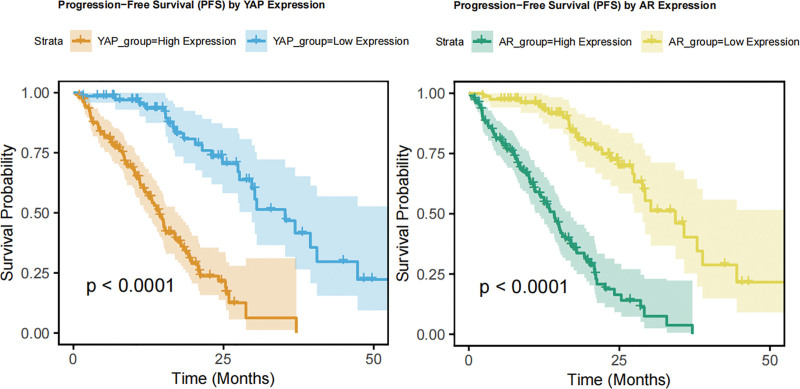
YAP and AR group high and low PFS. AR = androgen receptor, PFS = progression-free survival, YAP = Yes-associated protein.

High expression of YAP and AR was significantly associated with reduced PFS in patients with osteosarcoma (Table 4, Fig. 1). Univariate survival analysis showed that the median PFS in the YAP high expression group was 12 months, significantly shorter than the 28 months observed in the low expression group (HR = 3.20, 95% CI 2.00–5.10, *P* < .001). Similarly, the AR high expression group had a median PFS of 13.5 months, which was markedly shorter than the 25 months in the low expression group (HR = 2.90, 95% CI 1.80–4.60, *P* < .001). These findings indicate that high expression of YAP and AR are independent risk factors for rapid disease progression in osteosarcoma, suggesting that the two may accelerate tumor resistance or invasiveness through a synergistic mechanism.

**Table 4:** Univariate survival analysis for PFS.

Indicator	Group	Median survival time (mo)	HR (95% CI)	*P*-value
PFS				
YAP expression	High expression (n = 65)	12	3.20 (2.00–5.10)	<.001
	Low expression (n = 35)	28	1.00 (Reference)	
AR expression	High expression (n = 60)	13.5	2.90 (1.80–4.60)	<.001
	Low expression (n = 40)	25	1.00 (Reference)	

AR = androgen receptor, CI = confidence interval, HR = hazard ratio, PFS = progression-free survival, YAP = Yes-associated protein.

### 3.5. Co-expression of YAP and AR synergistically increases risk of disease progression

The combined expression of YAP and AR significantly intensified the risk of disease progression in osteosarcoma patients (Table 5). Joint survival analysis revealed that patients in the YAP⁺/AR⁺ group had a median PFS of only 9 months, with a 6.50-fold higher risk of disease progression compared to the YAP⁻/AR⁻ group (95% CI 3.20–13.20, *P* < .001).

**Table 5:** Relationship between combined YAP/AR expression and progression-free survival (PFS).

Combined expression group	Sample size (n)	Median PFS (mo)	HR (95% CI)	*P*-value (vs YAP-/AR-)
YAP+/AR+	45	9	6.50 (3.20–13.20)	<.001
YAP+/AR-	20	15	3.00 (1.50–6.00)	.002
YAP-/AR+	15	18	2.20 (1.00–4.80)	.051
YAP-/AR-	20	36	1.00	-

AR = androgen receptor, CI = confidence interval, HR = hazard ratio, PFS = progression-free survival, YAP = Yes-associated protein.

Even in cases of single-marker high expression (YAP⁺/AR⁻ or YAP⁻/AR⁺), the median PFS was still significantly shorter than in the dual low-expression group (15 months, HR = 3.00, *P* = .002; 18 months, HR = 2.20, *P* = .051). Notably, the prognostic risk associated with YAP high expression alone (YAP⁺/AR⁻) was greater than that of AR high expression alone (YAP⁻/AR⁺), suggesting that YAP may be the primary driver of disease progression. These results further confirm that the synergistic interaction between YAP and AR markedly amplifies the aggressive biological behavior of the tumor.

### 3.6. Interaction between YAP and AR significantly amplifies the risk of disease progression

Interaction analysis confirmed a significant synergistic oncogenic effect between YAP and AR in osteosarcoma (Table 6). In the Cox regression model, high expression of YAP alone increased the risk of disease progression by 2.80-fold (95% CI 1.70–4.60, *P* < .001), while AR high expression alone increased the risk by 2.20-fold (95% CI 1.40–3.50, *P* = .001). Notably, the interaction term (YAP × AR) further amplified this risk by an additional 1.80-fold (95% CI 1.20–2.70, *P* = .004), indicating that YAP and AR do not act independently but rather exacerbate malignant tumor progression through a synergistic mechanism. These findings suggest that co-targeting the YAP and AR signaling pathways may represent a promising strategy for improving prognosis in osteosarcoma patients.

**Table 6:** Interaction analysis between YAP and AR expression (PFS analysis).

Variable	HR (95% CI)	*P*-value
YAP high expression (alone)	2.80 (1.70–4.60)	<.001
AR high expression (alone)	2.20 (1.40–3.50)	.001
YAP × AR interaction	1.80 (1.20–2.70)	.004

AR = androgen receptor, CI = confidence interval, HR = hazard ratio, PFS = progression-free survival, YAP = Yes-associated protein.

## 4. Discussion

Through systematic immunohistochemical analysis and clinical follow-up, this study comprehensively explored the expression characteristics and clinical significance of YAP and AR in osteosarcoma, yielding several key findings. The following discussion provides an in-depth analysis of the results, along with the study’s limitations and directions for future research.

Firstly, we found that the expression levels of YAP and AR were significantly higher in osteosarcoma tissues compared to normal bone tissues (*P* < .001). This result is consistent with previous reports of YAP overexpression in various malignancies; however, the concurrent evaluation of YAP and AR expression in osteosarcoma is reported here for the first time. Notably, the high expression rate of YAP (65.0%) was slightly higher than that of AR (60.0%), which may reflect the more fundamental role of YAP as a core effector of the Hippo pathway in tumorigenesis. From a molecular perspective, YAP may provide a foundation for AR’s oncogenic activity by regulating processes such as cell proliferation and epithelial-mesenchymal transition, while AR may, in turn, enhance YAP’s oncogenic effects through modulation of transcriptional activity.^[12,13]^ The upregulation of YAP and AR in tumor tissues compared to normal controls suggests that these two molecules may jointly contribute to the pathogenesis of osteosarcoma, offering new insights into the molecular mechanisms underlying this disease.

Further analysis revealed a close association between high expression of YAP and AR and the clinical features of osteosarcoma, particularly in relation to advanced tumor stage and metastatic status. We observed that both YAP and AR were more frequently overexpressed in advanced-stage (Stage III–IV) and metastatic osteosarcomas, suggesting a potential role in driving tumor invasiveness and dissemination. The particularly high expression rates of YAP and AR in metastatic cases are in line with previous studies on other cancers.^[9,14]^ The involvement of YAP and AR in metastasis may be mediated through modulation of the tumor microenvironment and enhancement of tumor cell invasion and migration. Specifically, YAP may increase cellular invasiveness by promoting proliferation and survival, while AR may activate downstream signaling pathways that facilitate tumor cell growth and spread.^[15,16]^ Their synergistic interaction likely amplifies the malignant behavior of osteosarcoma.

Of particular note, the high expression rate of YAP was significantly greater in osteoblastic osteosarcoma compared to other subtypes (75.0% vs 50.0%, *P* = .02), providing new clues to the molecular characteristics of different pathological subtypes. Based on previous studies, we hypothesize that YAP may play a more prominent role in osteoblastic osteosarcoma by regulating genes involved in osteogenic differentiation, such as Runt-related transcription factor 2.^[4]^

In terms of survival analysis, we observed that high expression of YAP and AR significantly shortened PFS in osteosarcoma patients, further confirming the potential of YAP and AR as prognostic biomarkers. Notably, patients with concurrent high expression of YAP and AR exhibited the shortest PFS among all groups, suggesting that YAP and AR may accelerate tumor progression through a synergistic mechanism. Interaction analysis also revealed that the combined effect of YAP and AR significantly amplified the risk of disease progression, providing a theoretical basis for dual-targeted therapy against YAP and AR.^[3,17]^ In future therapeutic strategies, simultaneous inhibition of the YAP and AR signaling pathways may represent a promising direction for osteosarcoma treatment.

However, this study has several limitations that should be acknowledged. First, as a retrospective cohort study, it is inherently subject to case selection bias and information bias, since patient enrollment and data collection relied on existing medical records and follow-up information, which may not have been uniformly complete or accurate. Second, the single-center design and relatively small sample size may introduce regional and institutional bias, limiting the generalizability of our findings to broader populations. Third, although our results suggest a synergistic effect between YAP and AR in promoting osteosarcoma progression, this study did not include functional or molecular experiments to verify the underlying mechanisms, which constrains our ability to elucidate how YAP and AR interact and co-regulate tumor biology. Fourth, potential confounding factors, such as heterogeneity in surgical procedures and chemotherapy regimens, could not be fully controlled, which might have influenced survival outcomes. Fifth, the control tissues were obtained from areas more than 5 cm away from the tumor margin, which may not fully represent true normal bone due to possible microenvironmental influences. This approach is commonly adopted in osteosarcoma studies given the ethical and practical challenges of collecting healthy bone tissue. Future studies should aim to address these limitations by using prospective, multi-center designs with larger and more diverse cohorts, standardized treatment protocols, and validation in bone tissues from non-tumor patients, along with mechanistic experiments to confirm biological interactions.

In summary, this study reveals a close association between high expression of YAP and AR and the malignant progression of osteosarcoma. The co-overexpression of YAP and AR may serve as an important marker of poor prognosis and offer a potential tool for clinical prognostic evaluation. Moreover, therapeutic strategies targeting both YAP and AR may provide new avenues for osteosarcoma treatment. Despite certain limitations, this study lays an important foundation for understanding the molecular mechanisms of osteosarcoma and improving patient outcomes, opening new directions for future research and clinical application.

## 5. Conclusion

This study is the first to systematically evaluate the combined expression of YAP and AR in osteosarcoma. We found that their co-overexpression is closely associated with advanced stage, metastasis, and poor PFS, suggesting a synergistic role in driving tumor aggressiveness. Although the present work is based on immunohistochemistry and statistical associations without mechanistic validation, it provides important clinical evidence for the prognostic significance of YAP–AR co-expression in osteosarcoma. These findings warrant further functional studies to elucidate the molecular mechanisms underlying their interaction and to assess whether dual targeting of YAP and AR could represent a feasible therapeutic strategy in the future.

## Author contributions

**Conceptualization:** Juan Wang, Ping Xiang, Chuanqi Zou, Jun Wang.

**Data curation:** Juan Wang, Ping Xiang, Chuanqi Zou, Jun Wang.

**Formal analysis:** Juan Wang, Ping Xiang, Chuanqi Zou, Jun Wang.

**Funding acquisition:** Jun Wang.

**Investigation:** Juan Wang, Ping Xiang, Chuanqi Zou, Jun Wang.

**Methodology:** Juan Wang, Ping Xiang, Chuanqi Zou.

**Supervision:** Juan Wang, Ping Xiang, Chuanqi Zou, Jun Wang.

**Validation:** Juan Wang, Ping Xiang, Chuanqi Zou, Jun Wang.

**Visualization:** Juan Wang, Ping Xiang, Chuanqi Zou, Jun Wang.

**Writing – original draft:** Juan Wang, Ping Xiang, Chuanqi Zou, Jun Wang.

**Writing – review & editing:** Juan Wang, Chuanqi Zou, Jun Wang.
